# Some normed binomial difference sequence spaces related to the $\ell_{p}$ spaces

**DOI:** 10.1186/s13660-017-1401-4

**Published:** 2017-06-02

**Authors:** Meimei Song, Jian Meng

**Affiliations:** Department of Mathematics, Tianjin University of Technology, Tianjin, 300000 P.R. China

**Keywords:** sequence space, matrix domain, Schauder basis, *α*-, *β*- and *γ*-duals

## Abstract

The aim of this paper is to introduce the normed binomial sequence spaces $b^{r,s}_{p}(\nabla)$ by combining the binomial transformation and difference operator, where $1\leq p\leq\infty$. We prove that these spaces are linearly isomorphic to the spaces $\ell_{p}$ and $\ell _{\infty}$, respectively. Furthermore, we compute Schauder bases and the *α*-, *β*- and *γ*-duals of these sequence spaces.

## Introduction and preliminaries

Let *w* denote the space of all sequences. By $\ell_{p}$, $\ell_{\infty }$, *c* and $c_{0}$, we denote the spaces of *p*-absolutely summable, bounded, convergent and null sequences, respectively, where $1\leq p<\infty$. Let *Z* be a sequence space, then Kizmaz [1] introduced the following difference sequence spaces:

$$Z(\Delta)=\bigl\{ (x_{k})\in w:(\Delta x_{k})\in Z\bigr\} $$

for $Z=\ell_{\infty},c,c_{0}$, where $\Delta x_{k}=x_{k}-x_{k+1}$ for each $k\in\mathbb{N}=\{1,2,3,\ldots\}$, the set of positive integers. Since then, many authors have studied further generalization of the difference sequence spaces [2–6]. Moreover, Altay and Polat [7], Başarir and Kara [8–12], Kara [13], Kara and İlkhan [14], Polat and Başar [15], and many others have studied new sequence spaces from a matrix point of view that represent difference operators.

For an infinite matrix $A=(a_{n,k})$ and $x=(x_{k})\in w$, the *A*-transform of *x* is defined by $Ax=\{(Ax)_{n}\}$ and is supposed to be convergent for all $n\in\mathbb{N}$, where $(Ax)_{n}=\sum_{k=0}^{\infty}a_{n,k}x_{k}$. For two sequence spaces *X*, *Y* and an infinite matrix $A=(a_{n,k})$, the sequence space $X_{A}$ is defined by $X_{A}=\{x=(x_{k})\in w:Ax \in X\}$, which is called the domain of matrix *A* in the space *X*. By $(X: Y)$, we denote the class of all matrices such that $X \subseteq Y_{A}$.

The Euler means $E^{r}$ of order *r* is defined by the matrix $E^{r}=(e_{n,k}^{r})$, where $0< r<1$ and

$$e_{n,k}^{r}= \textstyle\begin{cases} \left({\scriptsize\begin{matrix}{} n\cr k \end{matrix}} \right)(1-r)^{n-k}r^{k}& \text{if $0\leq k\leq n$},\\ 0& \text{if $k>n$}. \end{cases} $$

The Euler sequence spaces $e^{r}_{p}$ and $e^{r}_{\infty}$ were defined by Altay, Başar and Mursaleen [16] as follows:

$$e^{r}_{p}=\left \{x=(x_{k})\in w: \sum _{n}\left \vert \sum _{k=0}^{n} \left ( \begin{matrix} n\\ k \end{matrix} \right ) (1-r)^{n-k}r^{k}x_{k} \right \vert ^{p}< \infty \right \} $$

and

$$e^{r}_{\infty}=\left \{x=(x_{k})\in w: \sup _{n\in\mathbb{N}}\left \vert \sum_{k=0}^{n} \left ( \begin{matrix} n\\ k \end{matrix} \right ) (1-r)^{n-k}r^{k}x_{k} \right \vert < \infty \right \}. $$

Altay and Polat [7] defined further generalization of the Euler sequence spaces $e^{r}_{0}(\nabla)$, $e^{r}_{c}(\nabla)$ and $e^{r}_{\infty}(\nabla)$ by

$$\begin{aligned} &e^{r}_{0}(\nabla)= \bigl\{ x=(x_{k})\in w: ( \nabla x_{k})\in e^{r}_{0} \bigr\} , \\ &e^{r}_{c}(\nabla)= \bigl\{ x=(x_{k})\in w: ( \nabla x_{k})\in e^{r}_{c} \bigr\} \end{aligned}$$

and

$$\begin{aligned} e^{r}_{\infty}(\nabla)= \bigl\{ x=(x_{k})\in w: (\nabla x_{k})\in e^{r}_{\infty} \bigr\} , \end{aligned}$$

where $\nabla x_{k}=x_{k}-x_{k-1}$ for each $k\in\mathbb{N}$. Here any term with negative subscript is equal to naught. Many authors have used especially the Euler matrix for defining new sequence spaces, for instance, Kara and Başarir [17], Karakaya and Polat [18] and Polat and Başar [15].

Recently Bişgin [19, 20] defined another type of generalization of the Euler sequence spaces and introduced the binomial sequence spaces $b^{r,s}_{0}$, $b^{r,s}_{c}$, $b^{r,s}_{\infty }$ and $b^{r,s}_{p}$. Let $r,s\in\mathbb{R}$ and $r+s\neq0$. Then the binomial matrix $B^{r,s}=(b_{n,k}^{r,s})$ is defined by

$$b_{n,k}^{r,s}= \textstyle\begin{cases} \frac{1}{(s+r)^{n}}\left({\scriptsize\begin{matrix}{} n\cr k\end{matrix}} \right)s^{n-k}r^{k}& \text{if $0\leq k\leq n$},\\ 0& \text{if $k>n$}, \end{cases} $$

for all $k,n\in\mathbb{N}$. For $sr>0$ we have

(i) $\Vert B^{r,s} \Vert <\infty$,
(ii) $\lim_{n\rightarrow\infty}b_{n,k}^{r,s}=0$ for each $k\in \mathbb{N}$,
(iii) $\lim_{n\rightarrow\infty}\sum_{k}b_{n,k}^{r,s}=1$.

Thus, the binomial matrix $B^{r,s}$ is regular for $sr>0$. Unless stated otherwise, we assume that $sr >0$. If we take $s+r =1$, we obtain the Euler matrix $E^{r}$. So the binomial matrix generalizes the Euler matrix. Bişgin [20] defined the following spaces of binomial sequences:

$$b^{r,s}_{p}=\left \{x=(x_{k})\in w: \sum _{n}\left \vert \frac{1}{(s+r)^{n}}\sum _{k=0}^{n}\left ( \begin{matrix} n\\ k \end{matrix} \right )s^{n-k}r^{k}x_{k} \right \vert ^{p}< \infty \right \} $$

and

$$b^{r,s}_{\infty}=\left \{x=(x_{k})\in w: \sup _{n\in\mathbb{N}}\left \vert \frac {1}{(s+r)^{n}}\sum _{k=0}^{n}\left ( \begin{matrix} n\\ k \end{matrix} \right )s^{n-k}r^{k}x_{k} \right \vert < \infty \right \}. $$

The main purpose of the present paper is to study the normed difference spaces $b^{r,s}_{p}(\nabla)$ and $b^{r,s}_{\infty}(\nabla)$ of the binomial sequence whose $B^{r,s}(\nabla)$-transforms are in the spaces $\ell_{p}$ and $\ell_{\infty}$, respectively. These new sequence spaces are the generalization of the sequence spaces defined in [7] and [20]. Also, we compute the bases and *α*-, *β*- and *γ*-duals of these sequence spaces.

## The binomial difference sequence spaces

In this section, we introduce the spaces $b^{r,s}_{p}(\nabla)$ and $b^{r,s}_{\infty}(\nabla)$ and prove that these sequence spaces are linearly isomorphic to the spaces $\ell_{p}$ and $\ell_{\infty}$, respectively.

We first define the binomial difference sequence spaces $b^{r,s}_{p}(\nabla)$ and $b^{r,s}_{\infty}(\nabla)$ by

$$b^{r,s}_{p}(\nabla)= \bigl\{ x=(x_{k})\in w: (\nabla x_{k})\in b^{r,s}_{p} \bigr\} $$

and

$$b^{r,s}_{\infty}(\nabla)= \bigl\{ x=(x_{k})\in w: (\nabla x_{k})\in b^{r,s}_{\infty} \bigr\} . $$

Let us define the sequence $y=(y_{n})$ as the $B^{r,s}(\nabla )$-transform of a sequence $x=(x_{k})$, that is,

2.1 $$ y_{n}= \bigl[B^{r,s}(\nabla x_{k}) \bigr]_{n}=\frac{1}{(s+r)^{n}}\sum_{k=0}^{n} \left ( \begin{matrix} n\\ k \end{matrix} \right )s^{n-k}r^{k}(\nabla x_{k}) $$

for each $n\in\mathbb{N}$. Then the binomial difference sequence spaces $b^{r,s}_{p}(\nabla)$ or $b^{r,s}_{\infty}(\nabla)$ can be redefined by all sequences whose $B^{r,s}(\nabla)$-transforms are in the space $\ell_{p}$ or $\ell_{\infty}$.

### Theorem 2.1

*The sequence spaces* $b^{r,s}_{p}(\nabla)$ *and* $b^{r,s}_{\infty}(\nabla )$ *are complete linear metric spaces with the norm defined by*

$$f_{b^{r,s}_{p}(\nabla)}(x)= \Vert y \Vert _{p}= \Biggl(\sum _{n=1}^{\infty} \vert y_{n} \vert ^{p} \Biggr)^{\frac{1}{p}} $$

*and*

$$f_{b^{r,s}_{\infty}(\nabla)}(x)= \Vert y \Vert _{\infty}=\sup_{n\in\mathbb{N}} \vert y_{n} \vert , $$

*where* $1\leq p<\infty$ *and the sequence* $y=(y_{n})$ *is defined by the* $B^{r,s}(\nabla)$-*transform of* *x*.

### Proof

The proof of the linearity is a routine verification. It is obvious that $f_{b^{r,s}_{p}}(\alpha x)= \vert \alpha \vert f_{b^{r,s}_{p}}(x)$ and $f_{b^{r,s}_{p}}(x)=0$ if and only if $x=\theta$ for all $x\in b^{r,s}_{p}(\nabla)$, where *θ* is the zero element in $b^{r,s}_{p}$ and $\alpha\in\mathbb{R}$. We consider $x,z \in b^{r,s}_{p}(\nabla)$, then we have

$$\begin{aligned} f_{b^{r,s}_{p}(\nabla)}(x+z)&= \biggl(\sum_{n} \bigl\vert \bigl(B^{r,s} \bigl[\nabla (x_{k}+z_{k}) \bigr] \bigr)_{n} \bigr\vert ^{p} \biggr)^{\frac{1}{p}} \\ &\leq \biggl(\sum_{n} \bigl\vert \bigl[B^{r,s}(\nabla x_{k}) \bigr]_{n} \bigr\vert ^{p} \biggr)^{\frac {1}{p}}+ \biggl(\sum_{n} \bigl\vert \bigl[B^{r,s}(\nabla z_{k}) \bigr]_{n} \bigr\vert ^{p} \biggr)^{\frac {1}{p}} = f_{b^{r,s}_{p}(\nabla)}(x)+f_{b^{r,s}_{p}(\nabla)}(z). \end{aligned}$$

Hence $f_{b^{r,s}_{p}(\nabla)}$ is a norm on the space $b^{r,s}_{p}(\nabla)$.

Let $(x_{m})$ be a Cauchy sequence in $b^{r,s}_{p}(\nabla)$, where $x_{m}=(x_{m_{k}})_{k=1}^{\infty}\in b^{r,s}_{p}(\nabla)$ for each $m\in\mathbb{N}$. For every $\varepsilon>0$, there is a positive integer $m_{0}$ such that $f_{b^{r,s}_{p}(\nabla)}( x_{m}-x_{l})<\varepsilon$ $\text{for } m,l\geq m_{0}$. Then we get

$$\bigl\vert \bigl(B^{r,s} \bigl[\nabla(x_{m_{k}}-x_{l_{k}}) \bigr] \bigr)_{n} \bigr\vert \leq \biggl(\sum _{n} \bigl\vert \bigl(B^{r,s} \bigl[ \nabla(x_{m_{k}}-x_{l_{k}}) \bigr] \bigr)_{n} \bigr\vert ^{p} \biggr)^{\frac {1}{p}}< \varepsilon $$

for $m,l\geq m_{0}$ and each $k\in\mathbb{N}$. So $(B^{r,s}(\nabla x_{m_{k}}))_{m=1}^{\infty}$ is a Cauchy sequence in the set of real numbers $\mathbb{R}$. Since $\mathbb{R}$ is complete, we have $\lim_{m\rightarrow\infty}B^{r,s}(\nabla x_{m_{k}})=B^{r,s}(\nabla x_{k})$ for each $k\in\mathbb{N}$. We compute

2.2 $$\begin{aligned} \sum_{n=0}^{i} \bigl\vert \bigl(B^{r,s} \bigl[\nabla(x_{m_{k}}-x_{l_{k}}) \bigr] \bigr)_{n} \bigr\vert \leq f_{b^{r,s}_{p}(\nabla)}( x_{m}-x_{l})< \varepsilon \end{aligned}$$

for $m>m_{0}$. We take *i* and *l* →∞, then the inequality (2.2) implies that

$$f_{b^{r,s}_{p}(\nabla)}( x_{m}-x)\rightarrow0. $$

We have

$$f_{b^{r,s}_{p}(\nabla)}(x)\leq f_{b^{r,s}_{p}(\nabla )}(x_{m}-x)+f_{b^{r,s}_{p}(\nabla)}(x_{m})< \infty, $$

that is, $x\in b^{r,s}_{p}(\nabla)$. Thus, the space $b^{r,s}_{p}(\nabla )$ is complete. For the space $b^{r,s}_{\infty}(\nabla)$, the proof can be completed in a similar way. So, we omit the detail. □

### Theorem 2.2

*The sequence spaces* $b^{r,s}_{p}(\nabla)$ *and* $b^{r,s}_{\infty}(\nabla )$ *are linearly isomorphic to the spaces* $\ell_{p}$ *and* $\ell_{\infty}$, *respectively*, *where* $1\leq p< \infty$.

### Proof

Similarly, we only prove the theorem for the space $b^{r,s}_{p}(\nabla)$. To prove $b^{r,s}_{p}(\nabla)\cong\ell _{p}$, we must show the existence of a linear bijection between the spaces $b^{r,s}_{p}(\nabla)$ and $\ell_{p}$.

Consider $T:b^{r,s}_{p}(\nabla)\rightarrow\ell_{p}$ by $T(x)=B^{r,s}(\nabla x_{k})$. The linearity of *T* is obvious and $x=\theta$ whenever $T(x)=\theta$. Therefore, *T* is injective.

Let $y=(y_{n})\in\ell_{p} $ and define the sequence $x=(x_{k})$ by

2.3 $$\begin{aligned} x_{k}=\sum_{i=0}^{k}(s+r)^{i} \sum_{j=i}^{k} \left ( \begin{matrix} j\\ i \end{matrix} \right )r^{-j}(-s)^{j-i}y_{i} \end{aligned}$$

for each $k \in\mathbb{N}$. Then we have

$$\begin{aligned} f_{b^{r,s}_{p}(\nabla)}(x) =& \bigl\Vert \bigl[B^{r,s}(\nabla x_{k}) \bigr]_{n} \bigr\Vert _{p} \\ =&\left (\sum_{n=1}^{\infty} \left \vert \frac{1}{(s+r)^{n}}\sum_{k=0}^{n} \left ( \begin{matrix} n\\ k \end{matrix} \right )s^{n-k}r^{k}( \nabla x_{k}) \right \vert ^{p} \right )^{\frac{1}{p}} \\ =& \Biggl(\sum_{n=1}^{\infty} \vert y_{n} \vert ^{p} \Biggr)^{\frac{1}{p}} = \Vert y \Vert _{p}< \infty, \end{aligned}$$

which implies that $x\in b^{r,s}_{p}(\nabla)$ and $T(x)=y$. Consequently, *T* is surjective and is norm preserving. Thus, $b^{r,s}_{p}(\nabla)\cong\ell_{p}$. □

## The Schauder basis and *α*-, *β*- and *γ*-duals

For a normed space $(X, \Vert \cdot \Vert )$, a sequence $\{ x_{k}:x_{k}\in X\}_{k\in\mathbb{N}}$ is called a *Schauder basis* [21] if for every $x\in X$, there is a unique scalar sequence $(\lambda_{k})$ such that $\Vert x-\sum_{k=0}^{n}\lambda _{k}x_{k} \Vert \rightarrow0 \text{ as } n\rightarrow\infty$. Next, we shall give a Schauder basis for the sequence space $b_{p}^{r,s}(\nabla)$.

We define the sequence $g^{(k)}(r,s)=\{g^{(k)}_{i}(r,s)\}_{i \in\mathbb {N}}$ by

$$g^{(k)}_{i}(r,s)= \textstyle\begin{cases} 0& \text{if $0\leq i < k$},\\ (s+r)^{k}\sum_{j=k}^{i}\bigl({\scriptsize\begin{matrix}{} j\cr k\end{matrix}} \bigr)r^{-j}(-s)^{j-k}& \text{if $i\geq k$}, \end{cases} $$

for each $k\in\mathbb{N}$.

### Theorem 3.1

*The sequence* $(g^{(k)}(r,s))_{k\in\mathbb{N}}$ *is a Schauder basis for the binomial sequence spaces* $b_{p}^{r,s}(\nabla)$ *and every* $x=(x_{i})\in b_{p}^{r,s}(\nabla)$ *has a unique representation by*

3.1 $$ x=\sum_{k} \lambda_{k}(r,s) g^{(k)}(r,s), $$

*where* $1\leq p<\infty$ *and* $\lambda_{k}(r,s)= [B^{r,s}(\nabla x_{i})]_{k}$ *for each* $k\in\mathbb{N}$.

### Proof

Obviously, $B^{r,s}(\nabla g^{(k)}_{i}(r,s))=e_{k}\in\ell_{p}$, where $e_{k}$ is the sequence with 1 in the *k*th place and zeros elsewhere for each $k\in\mathbb{N}$. This implies that $g^{(k)}(r,s)\in b_{p}^{r,s}(\nabla)$ for each $k\in\mathbb{N}$.

For $x \in b_{p}^{r,s}(\nabla)$ and $m\in\mathbb{N}$, we put

$$x^{(m)}=\sum_{k=0}^{m} \lambda_{k}(r,s) g^{(k)}(r,s). $$

By the linearity of $B^{r,s}(\nabla)$, we have

$$B^{r,s} \bigl(\nabla x^{(m)}_{i} \bigr)=\sum _{k=0}^{m}\lambda _{k}(r,s)B^{r,s} \bigl(\nabla g^{(k)}_{i}(r,s) \bigr)=\sum _{k=0}^{m}\lambda_{k}(r,s)e_{k} $$

and

$$\bigl[B^{r,s} \bigl(\nabla \bigl(x_{i}-x_{i}^{(m)} \bigr) \bigr) \bigr]_{k}= \textstyle\begin{cases} 0& \text{if $0\leq k \leq m$},\\ [B^{r,s}(\nabla x_{i})]_{k}& \text{if $k> m$}, \end{cases} $$

for each $k\in\mathbb{N}$.

For any given $\varepsilon>0$, there is a positive integer $m_{0}$ such that

$$\sum_{k=m_{0}+1}^{\infty} \bigl\vert \bigl[B^{r,s}(\nabla x_{i}) \bigr]_{k} \bigr\vert ^{p}< \biggl(\frac {\varepsilon}{2} \biggr)^{p} $$

for all $k\geq m_{0}$. Then we have

$$\begin{aligned} f_{b^{r,s}_{p}(\nabla)} \bigl( x-x^{(m)} \bigr) =& \Biggl(\sum _{k=m+1 }^{\infty} \bigl\vert \bigl[B^{r,s}(\nabla x_{i}) \bigr]_{k} \bigr\vert ^{p} \Biggr)^{\frac{1}{p}} \\ \leq& \Biggl(\sum_{k=m_{0}+1 }^{\infty} \bigl\vert \bigl[B^{r,s}(\nabla x_{i}) \bigr]_{k} \bigr\vert ^{p} \Biggr)^{\frac {1}{p}} \\ < & \frac{\varepsilon}{2}< \varepsilon, \end{aligned}$$

which implies that $x \in b_{p}^{r,s}(\nabla)$ is represented as (3.1).

To prove the uniqueness of this representation, we assume that

$$x=\sum_{k} \mu_{k}(r,s) g^{(k)}(r,s). $$

Then we have

$$\bigl[B^{r,s}(\nabla x_{i}) \bigr]_{k}=\sum _{k}\mu_{k}(r,s) \bigl[B^{r,s} \bigl(\nabla g^{(k)}_{i}(r,s) \bigr) \bigr]_{k}=\sum _{k}\mu_{k}(r,s) (e_{k})_{k}= \mu_{k}(r,s), $$

which is a contradiction with the assumption that $\lambda _{k}(r,s)=[B^{r,s}(\nabla x_{i})]_{k}$ for each $k \in\mathbb{N}$. This shows the uniqueness of this representation. □

### Corollary 3.2

*The sequence space* $b_{p}^{r,s}(\nabla)$ *is separable*, *where* $1\leq p<\infty$.

For the duality theory, the study of sequence spaces is more useful when we investigate them equipped with linear topologies. Köthe and Toeplitz [22] first computed duals whose elements can be represented as sequences and defined the *α*-dual (or Köthe-Toeplitz dual).

For the sequence spaces *X* and *Y*, define the multiplier space $M(X,Y)$ by

$$M(X,Y)= \bigl\{ u=(u_{k})\in w:ux=(u_{k}x_{k})\in Y \text{ for all } x=(x_{k})\in X \bigr\} . $$

Then the *α*-, *β*- and *γ*-duals of a sequence space *X* are defined by

$$X^{\alpha}=M(X,\ell_{1}),\qquad X^{\beta}=M(X,c)\quad \text{and}\quad X^{\gamma }=M(X,\ell_{\infty}), $$

respectively.

We give the following properties:

3.2 $$\begin{aligned} &\sup_{n\in\mathbb{N}} \sum_{k} \vert a_{n,k} \vert ^{q}< \infty, \end{aligned}$$

3.3 $$\begin{aligned} &\sup_{k\in\mathbb{N}} \sum_{n} \vert a_{n,k} \vert < \infty, \end{aligned}$$

3.4 $$\begin{aligned} &\sup_{n,k\in\mathbb{N}} \vert a_{n,k} \vert < \infty, \end{aligned}$$

3.5 $$\begin{aligned} &\lim_{n\rightarrow\infty}a_{n,k}=a_{k} \quad\text{for each } k \in \mathbb{N}, \end{aligned}$$

3.6 $$\begin{aligned} &\sup_{K\in\Gamma} \sum_{k} \biggl\vert \sum_{n\in K} a_{n,k} \biggr\vert ^{q}< \infty , \end{aligned}$$

3.7 $$\begin{aligned} &\lim_{n\rightarrow\infty}\sum_{k} \vert a_{n,k} \vert =\sum_{k} \Bigl\vert \lim _{n\rightarrow\infty}a_{n,k} \Bigr\vert , \end{aligned}$$

where Γ is the collection of all finite subsets of $\mathbb{N}$, $\frac{1}{p}+\frac{1}{q}=1$ and $1< p\leq\infty$.

### Lemma 3.3

[23]

*Let* $A=(a_{n,k})$ *be an infinite matrix*. *Then the following statements hold*:

(i) $A\in(\ell_{1}:\ell_{1})$ *if and only if* (3.3) *holds*.
(ii) $A\in(\ell_{1}:c)$ *if and only if* (3.4) *and* (3.5) *hold*.
(iii) $A\in(\ell_{1}:\ell_{\infty})$ *if and only if* (3.4) *holds*.
(iv) $A\in(\ell_{p}:\ell_{1})$ *if and only if* (3.6) *holds with* $\frac {1}{p}+\frac{1}{q}=1$ *and* $1< p\leq\infty$.
(v) $A\in( \ell_{p}:c)$ *if and only if* (3.2) *and* (3.5) *hold with* $\frac{1}{p}+\frac{1}{q}=1$ *and* $1< p<\infty$.
(vi) $A\in( \ell_{p}:\ell_{\infty} )$ *if and only if* (3.2) *holds with* $\frac{1}{p}+\frac{1}{q}=1$ *and* $1< p<\infty$.
(vii) $A\in( \ell_{\infty}:c )$ *if and only if* (3.5) *and* (3.7) *hold with* $\frac{1}{p}+\frac{1}{q}=1$ *and* $1< p<\infty$.
(viii) $A\in( \ell_{\infty}:\ell_{\infty} )$ *if and only if* (3.2) *holds with* $q=1$.

### Theorem 3.4

*We define the set* $U_{1}^{r,s}$ *and* $U_{2}^{r,s}$ *by*

$$U_{1}^{r,s}=\left \{u=(u_{k})\in w:\sup _{i\in\mathbb{N}}\sum_{k}\left \vert (s+r)^{i}\sum_{j=i}^{k}\left ( \begin{matrix} j\\ i \end{matrix} \right )r^{-j}(-s)^{j-i}u_{k} \right \vert < \infty \right \} $$

*and*

$$U_{2}^{r,s}=\left \{u=(u_{k})\in w:\sup _{K\in\Gamma}\sum_{i}\left \vert \sum_{k\in K}(s+r)^{i}\sum _{j=i}^{k}\left ( \begin{matrix} j\\ i \end{matrix} \right )r^{-j}(-s)^{j-i}u_{k} \right \vert ^{q}< \infty \right \}. $$

*Then* $[b^{r,s}_{1}(\nabla)]^{\alpha}=U_{1}^{r,s}$ *and* $[b^{r,s}_{p}(\nabla)]^{\alpha}=U_{2}^{r,s}$, *where* $1< p\leq\infty$.

### Proof

Let $u=(u_{k})\in w$ and $x=(x_{k})$ be defined by (2.3), then we have

$$u_{k}x_{k}=\sum_{i=0}^{k}(s+r)^{i} \sum_{j=i}^{k}\left ( \begin{matrix} j\\ i \end{matrix} \right )r^{-j}(-s)^{j-i}u_{k}y_{i}= \bigl(G^{r,s}y \bigr)_{k} $$

for each $k\in\mathbb{N}$, where $G^{r,s}=(g^{r,s}_{k,i})$ is defined by

$$g^{r,s}_{k,i}= \textstyle\begin{cases} (s+r)^{i}\sum_{j=i}^{k}\left({\scriptsize\begin{matrix}{} j\cr i \end{matrix}} \right)r^{-j}(-s)^{j-i}u_{k}& \text{if $0\leq i\leq k$},\\ 0& \text{if $i>k$}. \end{cases} $$

Therefore, we deduce that $ux= (u_{k}x_{k})\in\ell_{1}$ whenever $x\in b_{1}^{r,s}(\nabla)$ or $b_{p}^{r,s}(\nabla)$ if and only if $G^{r,s}y\in\ell_{1}$ whenever $y\in \ell_{1}$ or $\ell_{p}$, which implies that $u=(u_{k})\in[b_{1}^{r,s}(\nabla )]^{\alpha} \text{ or } [b_{p}^{r,s}(\nabla)]^{\alpha}$ if and only if $G^{r,s}\in(\ell_{1}:\ell_{1})$ and $G^{r,s}\in(\ell_{p}:\ell_{1})$ by parts (i) and (iv) of Lemma 3.3, we obtain $u=(u_{k})\in [b_{1}^{r,s}(\nabla)]^{\alpha}$ if and only if

$$\sup_{i\in\mathbb{N}}\sum_{k}\left \vert (s+r)^{i}\sum_{j=i}^{k} \left ( \begin{matrix} j\\ i \end{matrix} \right )r^{-j}(-s)^{j-i}u_{k} \right \vert < \infty $$

and $u=(u_{k})\in[b_{p}^{r,s}(\nabla)]^{\alpha}$ if and only if

$$\sup_{K\in\Gamma}\sum_{i}\left \vert \sum_{k\in K}(s+r)^{i}\sum _{j=i}^{k}\left ( \begin{matrix} j\\ i \end{matrix} \right )r^{-j}(-s)^{j-i}u_{k} \right \vert ^{q}< \infty. $$

Thus, we have $[b^{r,s}_{1}(\nabla)]^{\alpha}=U_{1}^{r,s}$ and $[b^{r,s}_{p}(\nabla)]^{\alpha}=U_{2}^{r,s}$, where $1< p\leq\infty$. □

Now, we define the sets $U_{3}^{r,s}$, $U_{4}^{r,s}$, $U_{5}^{r,s}$, $U_{6}^{r,s}$ and $U_{7}^{r,s}$ by

$$\begin{aligned} &U_{3}^{r,s}=\left \{u=(u_{k})\in w: \lim _{n\rightarrow\infty} (s+r)^{k}\sum_{i=k}^{n} \sum_{j=k}^{i}\left ( \begin{matrix} j\\ k \end{matrix} \right )r^{-j}(-s)^{j-k}u_{i} \text{ exists for each } k \in\mathbb {N} \right \}, \\ &U_{4}^{r,s}=\left \{u=(u_{k})\in w: \sup _{n,k\in\mathbb{N}}\left \vert (s+r)^{k}\sum _{i=k}^{n}\sum_{j=k}^{i} \left ( \begin{matrix} j\\ k \end{matrix} \right )r^{-j}(-s)^{j-k}u_{i} \right \vert < \infty \right \}, \\ &U_{5}^{r,s}= \left\{u=(u_{k})\in w: \lim _{n\rightarrow\infty}\sum_{k}\left \vert (s+r)^{k}\sum_{i=k}^{n}\sum _{j=k}^{i}\left ( \begin{matrix} j\\ k \end{matrix} \right )r^{-j}(-s)^{j-k}u_{i} \right \vert \right. \\ &\left.\phantom{U_{5}^{r,s}=}=\sum_{k}\left \vert \lim _{n\rightarrow\infty}(s+r)^{k}\sum_{i=k}^{n} \sum_{j=k}^{i}\left ( \begin{matrix} j\\ k \end{matrix} \right )r^{-j}(-s)^{j-k}u_{i} \right \vert \right\}, \\ &U_{6}^{r,s}=\left \{u=(u_{k})\in w: \sup _{n\in\mathbb{N}}\sum_{k=0}^{n}\left \vert (s+r)^{k}\sum_{i=k}^{n} \sum_{j=k}^{i}\left ( \begin{matrix} j\\ k \end{matrix} \right )r^{-j}(-s)^{j-k}u_{i} \right \vert ^{q}< \infty \right \},\quad 1< q< \infty, \end{aligned}$$

and

$$U_{7}^{r,s}=\left \{u=(u_{k})\in w: \sup _{n\in\mathbb{N}}\sum_{k=0}^{n}\left \vert (s+r)^{k}\sum_{i=k}^{n} \sum_{j=k}^{i}\left ( \begin{matrix} j\\ k \end{matrix} \right )r^{-j}(-s)^{j-k}u_{i} \right \vert < \infty \right \}. $$

### Theorem 3.5

*We have the following relations*:

(i) $[b_{1}^{r,s}(\nabla)]^{ \beta}=U_{3}^{r,s}\cap U_{4}^{r,s}$,
(ii) $[b_{p}^{r,s}(\nabla)]^{ \beta}=U_{3}^{r,s}\cap U_{6}^{r,s}$, *where* $1< p<\infty$,
(iii) $[b_{\infty}^{r,s}(\nabla)]^{ \beta}=U_{3}^{r,s}\cap U_{5}^{r,s}$,
(iv) $[b_{1}^{r,s}(\nabla)]^{ \gamma}=U_{4}^{r,s}$,
(v) $[b_{p}^{r,s}(\nabla)]^{ \gamma}=U_{6}^{r,s}$, *where* $1< p<\infty $,
(vi) $[b_{\infty}^{r,s}(\nabla)]^{ \gamma}=U_{7}^{r,s}$.

### Proof

Let $u=(u_{k})\in w$ and $x=(x_{k})$ be defined by (2.3), then we consider the following equation:

$$\begin{aligned} \sum_{k=0}^{n}u_{k}x_{k}&= \sum_{k=0}^{n}u_{k}\left [ \sum_{i=0}^{k}(s+r)^{i}\sum _{j=i}^{k}\left ( \begin{matrix} j\\ i \end{matrix} \right )r^{-j}(-s)^{j-i}y_{i} \right ] \\ &=\sum_{k=0}^{n}\left [(s+r)^{k} \sum_{i=k}^{n}\sum _{j=k}^{i}\left ( \begin{matrix} j\\ k \end{matrix} \right )r^{-j}(-s)^{j-k}u_{i} \right ]y_{k} = \bigl(U^{r,s}y \bigr)_{n}, \end{aligned}$$

where $U^{r,s}=(u^{r,s}_{n,k})$ is defined by

$$u_{n,k}= \textstyle\begin{cases} (s+r)^{k}\sum_{i=k}^{n}\sum_{j=k}^{i}\bigl({\scriptsize\begin{matrix}{} j\cr k\end{matrix}} \bigr)r^{-j}(-s)^{j-k}u_{i}&\text{if $0\leq k \leq n$},\\ 0&\text{if $k> n$}. \end{cases} $$

Therefore, we deduce that $ux= (u_{k}x_{k})\in c$ whenever $x\in b_{1}^{r,s}(\nabla)$ if and only if $U^{r,s}y\in c$ whenever $y\in\ell_{1}$, which implies that $u=(u_{k})\in[b_{1}^{r,s}(\nabla)]^{ \beta}$ if and only if $U^{r,s}\in(\ell_{1}:c)$. By Lemma 3.3(ii), we obtain $[b_{1}^{r,s}(\nabla)]^{ \beta}=U_{3}^{r,s}\cap U_{4}^{r,s}$. Using Lemma 3.3(i) and (iii)-(viii) instead of (ii), the proof can be completed in a similar way. So, we omit the details. □

## Conclusion

By considering the definitions of the binomial matrix $B^{r,s}=(b^{r,s}_{n,k})$ and the difference operator, we introduce the sequence spaces $b^{r,s}_{p}(\nabla)$ and $b^{r,s}_{\infty}(\nabla)$. These spaces are the natural continuations of [1, 7, 20]. Our results are the generalizations of the matrix domain of the Euler matrix of order *r*. In order to give fully inform the reader on related topics with applications and a possible line of further investigation, the e-book [24] is added to the list of references.

## References

[1] Kizmaz H (1981). On certain sequence spaces. Can. Math. Bull..

[2] Bektaş C, Et M, Çolak R (2004). Generalized difference sequence spaces and their dual spaces. J. Math. Anal. Appl..

[3] Dutta H (2009). Characterization of certain matrix classes involving generalized difference summability spaces. Appl. Sci..

[4] Reddy BS (2010). On some generalized difference sequence spaces. Soochow J. Math..

[5] Tripathy BC, Esi A (2005). On a new type of generalized difference Cesàro sequence spaces. Soochow J. Math..

[6] Tripathy BC, Esi A (2006). A new type of difference sequence spaces. Int. J. Sci. Technol..

[7] Altay B, Polat H (2006). On some new Euler difference sequence spaces. Southeast Asian Bull. Math..

[8] Başarir M, Kara EE (2011). On compact operators on the Riesz ${B}^{m}$-difference sequence spaces. Iran. J. Sci. Technol..

[9] Başarir M, Kara EE (2011). On some difference sequence spaces of weighted means and compact operators. Ann. Funct. Anal..

[10] Başarir M, Kara EE (2012). On compact operators on the Riesz ${B}^{m}$-difference sequence spaces II. Iran. J. Sci. Technol..

[11] Başarir M, Kara EE (2012). On the B-difference sequence space derived by generalized weighted mean and compact operators. J. Math. Anal. Appl..

[12] Başarir M, Kara EE (2013). On the *m*th order difference sequence space of generalized weighted mean and compact operators. Acta Math. Sci..

[13] Kara EE (2013). Some topological and geometrical properties of new Banach sequence spaces. J. Inequal. Appl..

[14] Kara EE, İlkhan M (2015). On some Banach sequence spaces derived by a new band matrix. Br. J. Math. Comput. Sci..

[15] Polat H, Başar F (2007). Some Euler spaces of difference sequences of order *m*. Acta Math. Sci..

[16] Altay B, Başar F, Mursaleen M (2006). On the Euler sequence spaces which include the spaces $\ell_{p}$ and $\ell_{\infty}$ I. Inf. Sci..

[17] Kara EE, Başarir M (2011). On compact operators and some Euler ${B}^{(m)}$-difference sequence spaces. J. Math. Anal. Appl..

[18] Karakaya V, Polat H (2010). Some new paranormed sequence spaces defined by Euler and difference operators. Acta Sci. Math..

[19] Bişgin MC (2016). The binomial sequence spaces of nonabsolute type. J. Inequal. Appl..

[20] Bişgin MC (2016). The binomial sequence spaces which include the spaces $\ell_{p}$ and $\ell_{\infty}$ and geometric properties. J. Inequal. Appl..

[21] Choudhary B, Nanda S (1989). Functional Analysis with Application.

[22] Köthe G, Toeplitz O (1934). Linear Raume mit unendlichvielen koordinaten and Ringe unenlicher Matrizen. J. Reine Angew. Math..

[23] Stieglitz M, Tietz H (1977). Matrixtransformationen von folgenräumen eine ergebnisubersict. Math. Z..

[24] Başar F (2012). Summability Theory and its Applications.

